# Congestive heart failure

**Published:** 2019-03-13

**Authors:** Xuan Zhao

**Affiliations:** 1 University of British Columbia, British Columbia, Canada

**Figure:**
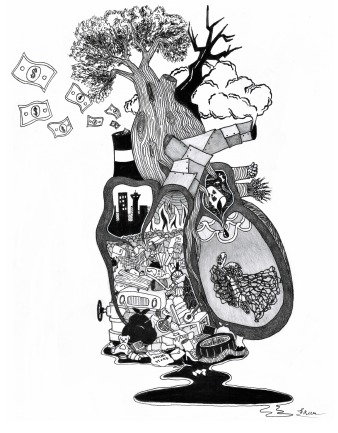


I felt an urge to create a piece that depicted the current state of our natural world using an analogy from medicine. The impact we have had on the natural environment has been like a disease. Current economies prioritize consumerism and expansion, congesting our world (the heart) with garbage and infecting our air, soil, and water with waste products. The belief we have a “right” to exploit the earth (right heart) has led to the rapid deterioration of what is “left” of the natural world (left heart).

Like congestive heart failure, we currently have no single cure for climate change, but that doesn't mean we can't create solutions for the future. In the words of David Attenborough at the 2019 World Economic Forum: “We need to move beyond guilt or blame, and get on with the practical tasks at hand. If people can truly understand what is at stake, I believe they will give permission for business and governments to get on with the practical solutions. And as a species, we are expert problem solvers. but we’ve not yet applied ourself to this problem with the focus that it requires. We can create a world with clean air and water, unlimited energy, and fish stocks that will sustain us well into the future. But to do that, we need a plan.”

## About the author

Xuan (Jingxuan) Zhao is a 2nd year medical student at UBC. For her, art is a medium for personal expression, advocacy, and exploration, be it through visual or performing arts. It’s also a nice escape from lectures and studying! She prefers being outdoors among the trees, but making art is one of the few things that can keep her indoors for hours (even days!) on end.

